
JOURNAL INFORMATION
==============================
NLM Title Abbreviation: ScientificWorldJournal
Journal ID: ScientificWorldJournal
NLM Journal ID: 101131163
Journal ID: TSWJ

The Scientific World Journal

ISSN: 2356-6140
EISSN: 1537-744X
Publisher: Wiley

ARTICLE INFORMATION
==============================
PMCID: PMC5763946
PMID: 21170485
DOI: 10.1100/tsw.2010.240
Article ID: 840583
Article version: 1
Subjects: Editorial

Pre- and Postnatal Management of Hydronephrosis

Hodges Steve J. *
Department of Urology, Wake Forest University School of Medicine, Winston-Salem, NC, USA
*Steve J. Hodges: shodges@wfubmc.eduAcademic Editor: Anthony Atala

Publication date: 2010
Electronic publication date: 2010 Dec 14
Volume: 10
First page: 2350
Last page: 2351
Received 2010 Dec 2; Accepted 2010 Dec 3
Copyright: Copyright © 2010 Steve J. Hodges.
Copyright year: 2010
License: This is an open access article distributed under the Creative Commons Attribution License, which permits unrestricted use, distribution, and reproduction in any medium, provided the original work is properly cited.
License URL: https://creativecommons.org/licenses/by/3.0/

==============================
Editorial
Special Issue: Pre- and Postnatal Management of Hydronephrosis
*TheScientificWorldJOURNAL* (2010) 10, 2350–2351
TSW Urology
ISSN 1537-744X; DOI 10.1100/tsw.2010.240

TheScientificWorldJOURNAL
www.thescientificworld.com

### Pre- and Postnatal Management of Hydronephrosis

Steve J. Hodges
*Department of Urology, Wake Forest University School of Medicine, Winston-Salem, NC*

**E-mail:** shodges@wfubmc.edu

*Received December 2, 2010; Accepted December 3, 2010; Published December 14, 2010*

---

Antenatal hydronephrosis is one of the most commonly identified anomalies on prenatal ultrasound examination. In fact, 1–5% of fetal ultrasounds will have abnormal collecting system dilation. This dilation represents a diagnostic dilemma, as it may represent urinary tract obstruction, reflux, or a normal state of development presenting as transient hydronephrosis. It follows that while it is true that the ubiquitous nature of modern antenatal sonography has increased our ability to detect urinary tract pathology (whether it is obstruction or reflux), decreasing the number of children who present later in life with symptomatic complications (such as hematuria, infections, or pain), the sheer number of antenatal tests being performed now has also increased the number of false-positive studies, forcing physicians to make distinctions between what they believe to be clinically significant and insignificant findings[1,2,3].

Further confounding the topic of antenatal hydronephrosis is the fact that there is little agreement on the degree of urinary tract dilation that signifies true antenatal hydronephrosis. Also, there is no evidence that early detection changes clinical outcomes for the better. Finally, even true hydronephrosis due to clinically identifiable causes may require little intervention and often resolves on its own, adding yet another clinical dilemma to the already muddied waters[1,2,4].

A recent consensus document from nine international medical centers reviewed over 400 articles related to antenatal hydronephrosis in an attempt to reach some agreement on these confusing issues. Unfortunately, due to the lack of prospective randomized controlled trials in the field, the group could only make general recommendation based on the current evidence[2].

A prime example of the difficulties inherent in the evaluation of antenatal hydronephrosis is that the definition of true hydronephrosis varies from study to study. There is an agreement that the anterior-posterior diameter of the renal pelvis in the transverse plane should be the standard measurement for assessing hydronephrosis, but the diameter that defines abnormal dilation significant of urinary tract pathology ranges from 7 to 15 mm depending on the author. One thing is certain, however, lower cutoffs increase the tests' sensitivity at the expense of specificity, while larger cutoffs do the exact opposite[1,2,3,5].

Other studies have added further detail to the antenatal ultrasound in an attempt to increase the ability of the test to pick up the truly significant cases of hydronephrosis. For example, the appearance of the renal parenchyma, the presence or absence of cysts, the bladder architecture, and the variation of hydronephrosis over repeated, spaced antenatal exams have all helped to increase our ability to detect true urinary tract pathology. Unfortunately, despite all these advancements, most children with antenatal hydronephrosis (regardless of their prenatal findings) receive similar postnatal evaluations to specifically define their disease process, if any. There is a hope that as technology improves, our ability to predict which children with antenatal hydronephrosis will require intervention will as well[2].

©2010 with author.
Published by TheScientificWorld; www.thescientificworld.com

There are many possible causes of antenatal hydronephrosis: from benign transient hydronephrosis, to ureteropelvic junction obstruction, vesicoureteral reflux, ureterovesical junction obstruction, multicystic dysplastic kidney, and posterior urethral valves; and many tools to diagnose and treat these disorders. This special issue on "Pre- and Postnatal Management of Hydronephrosis" of *TheScientificWorldJOURNAL* focuses on many of these causes, as well as the diagnostic dilemmas inherent in the diagnosis.

Rao and Palmer describe the general use of ultrasonography to diagnose antenatal hydronephrosis and how a differential diagnosis is developed for these findings. They detail prenatal parental counseling as well as postnatal evaluation of antenatal anomalies[4].

Kitchens and Herndon detail the diagnosis of the rare cases of severe antenatal hydronephrosis associated with oligohydramnios and the controversial interventions available to treat these difficult cases. They also provide a more detailed assessment of the postnatal evaluation of antenatal hydronephrosis, describing a systematic review of the upper and lower urinary tract, as well as the various imaging modalities available for infant evaluation[6,7].

Hodges et al.[8,9] and Herndon and Kitchens[10] then review some of the most common causes of antenatal hydronephrosis, including their diagnosis and treatment, such as ureteropelvic junction obstruction, megaureter, and posterior urethral valves.

The definitive management of antenatal hydronephrosis remains elusive, as there is little Level 1 evidence supporting the current strategies. However, our increased ability for early detection and our growing knowledge of the natural histories of the various causative disorders has greatly improved the care of children over the past few decades. This special issue summarizes some of the state-of-the-art recommendations regarding fetal hydronephrosis as we eagerly await more evidence-based guidelines.

### REFERENCES

1. Kitchens, D.M. and Herndon, C.D. (2009) Antenatal hydronephrosis. *Curr. Urol. Rep.* **10,** 126–133.
2. Nguyen, H.T., Herndon, C.D., Cooper, C., Gatti, J., Kirsch, A., Kokorowski, P., Lee, R., Perez-Brayfield, M., Metcalfe, P., Yerkes, E., Cendron, M., and Campbell, J.B. (2010) The Society for Fetal Urology consensus statement on the evaluation and management of antenatal hydronephrosis. *J. Pediatr. Urol.* **6,** 212–231.
3. Pates, J.A. and Dashe, J.S. (2006) Prenatal diagnosis and management of hydronephrosis. *Early Hum. Dev.* **82,** 3–8.
4. Rao, P.K. and Palmer, J.S. (2009) Prenatal and postnatal management of hydronephrosis. *TheScientificWorldJOURNAL* **9,** 606–614.
5. Herndon, C.D. (2006) Antenatal hydronephrosis: differential diagnosis, evaluation, and treatment options. *TheScientificWorldJOURNAL* **6,** 2345–2365.
6. Kitchens, D.M., and Herndon, C.D. (2009) Prenatal intervention for lower urinary tract obstruction. *TheScientificWorldJOURNAL* **9,** 390–392.
7. Kitchens, D.M., and Herndon, C.D. (2009) Postnatal imaging of antenatal hydronephrosis. *TheScientificWorldJOURNAL* **9,** 393–399.
8. Hodges, S.J., Patel, B., McLorie, G., and Atala, A. (2009) Posterior urethral valves. *TheScientificWorldJOURNAL* **9,** 1119–1126.
9. Hodges, S.J., Werle, D., McLorie, G., and Atala, A. Megaureter. *TheScientificWorldJOURNAL* **10,** 603–612.
10. Herndon, C.D., and Kitchens, D.M. (2009) The management of ureteropelvic junction obstruction presenting with prenatal hydronephrosis. *TheScientificWorldJOURNAL* **9,** 400–403.

**This article should be cited as follows:**

Hodges, S.J. (2010) Pre- and postnatal management of hydronephrosis. *TheScientificWorldJOURNAL:* TSW Urology **10**, 2350–2351. DOI 10.1100/tsw.2010.240.
